# Evaluating the Prevalence of Nasolacrimal Canal Injury in Midfacial Fractures: A Retrospective CT-Based Study

**DOI:** 10.7759/cureus.66161

**Published:** 2024-08-05

**Authors:** Mahima Seetaram, Devanshu Sinha, Karthik Ramakrishnan, Vivek N

**Affiliations:** 1 Department of Oral and Maxillofacial Surgery, SRM Kattankulathur Dental College and Hospital, SRM Institute of Science and Technology, Kattankulathur, IND

**Keywords:** computed tomography (ct ), surgery, midface, trauma, nasolacrimal

## Abstract

Introduction: Midface injuries, which are most common, can result in affectations to adjacent structures, including the nasolacrimal apparatus (NLA), which consists of the lacrimal sac, canaliculi, and nasolacrimal duct.

Objectives: The objectives of this study were to visualise the radiographic patency of the nasolacrimal canal in a computed tomography (CT) scan and assess the type of injury.

Methodology: This was a retrospective study wherein 322 CT scans of facial bones were analysed of patients who presented with midface fractures to the Department of Oral and Maxillofacial Surgery. The bony nasolacrimal canal was visualised on axial and coronal sections and confirmed on sagittal sections. The collapse of the bony nasolacrimal canal was measured using the advanced tools setting in the RadiAnt DICOM Viewer (Medixant, Poznań, Poland). The values were tabulated and statistically analysed.

Results: The incidence of NLA involvement in midface fractures was 37.6% (121 out of 322 fractures). The maximal involvement was seen in zygomaticomaxillary complex (ZMC) fractures. The visualisation of the fractures in the CT scan revealed that avulsion of the fossa was seen in 2.5% (eight out of 322 fractures), communication of the fossa or canal in 2.8% (nine out of 322 fractures), and linear fracture of the canal in 32.0% (103 out of 322 fractures). When measured in axial section, the Le Fort III fractures presented with a median of 2.15 mm, naso-orbito-ethmoid (NOE) fractures with a median of 0.90 mm, and fronto-naso-orbito-ethmoid (FNOE) fractures with a median of 1.15 mm. In the coronal section, the type of injuries that showed avulsion of the fossa had a median of 9.00 mm, communication of the fossa or canal showed 6.52 mm and linear fracture of the canal showed a median of 7.00 mm.

Conclusion: Many a time, the NLA is often neglected during a routine radiographic assessment of a CT scan in patients presenting with maxillofacial injuries. These injuries may not be clinically evident during examination. This might lead to postoperative clinical presentations in a patient. This study shows the various types of injuries to the NLA and its appearance on a CT scan. It also explains the requirement of soft tissue management and the clinical co-relationship of these injuries.

## Introduction

The median orbit and lateral nose comprise the bony nasolacrimal fossa and canal, which protect portions of the lacrimal apparatus [1,2].

The upper and lower eyelids each have a punctum in the eyelid margin near the medial canthus that drains into the lacrimal sac and the nasal cavity. This drainage canal connecting the ocular surface to the nasal cavity consists of multiple parts [1-3].

Midface injuries are common in trauma patients and can involve fractures of the zygomaticomaxillary complex (ZMC), Le Fort segments, nasal bones, and/or naso-orbito-ethmoid (NOE) complex [4]. These injuries can result in an undesirable impact on adjacent structures, including the nasolacrimal apparatus (NLA), which consists of the lacrimal sac, canaliculi, and nasolacrimal duct. The NLA is responsible for draining tears from the eye into the nasal cavity, and injury to this system can lead to epiphora, dacryocystitis, and other complications [4-6].

There is not much literature backup that describes fractures of the bony NLA in patients with facial trauma. Characterisation of these injuries may help maxillofacial surgeons better predict which patients will develop epiphora and dacryocystitis and who may eventually require surgical intervention. Understanding the incidence and nature of NLA injuries can help guide management decisions and improve outcomes. By identifying the frequency and patterns of NLA injury in this patient population, the recognition and management of this often overlooked complication is a potential subject for further understanding [1, 4-7].

The complications could be due to a lack of appropriate analysis of the NLA that interferes with its functioning. Though immediate anatomic reduction and functional aspects in relation to the fractured segment are obtained, the NLA is most commonly overlooked [6,8-11].

Computed tomography (CT) is commonly used to evaluate midface injuries and can also provide valuable information about the integrity of the NLA [9-11].

This article aimed to determine the incidence of NLA injury in patients with midface fractures, specifically ZMC, Le Fort segments, NOE complex, and nasal bone fractures. The CT scans of the participants were reviewed and assessed the NLA for signs of injury, including discontinuity or obstruction.

## Materials and methods

This retrospective analytical CT study was carried out in the Department of Oral and Maxillofacial Surgery of SRM Kattankulathur Dental College and Hospital, SRM Institute of Science and Technology, Kattankulathur, India. The study protocol was approved by the institutional ethical committee.

Three hundred and twenty-two CT scans of facial bones involving midfacial fractures were taken into consideration for those in age groups ranging from 18 to 70 years. Patients with previously treated fractures and artefacts on CT were excluded. Each CT was viewed in the available axial, coronal, and sagittal sections. The intraosseous component of the nasolacrimal apparatus (the bony canal and its margins) was assessed, and any discontinuity, collapse, or associated fractures were noted and measured.

In the CT, visualisation of the nasolacrimal canal, type of injury, measurement of injury from a common bony prominence, and level of injury were considered.

In the axial section, the nasolacrimal canal generally appears as a bony window wherein the lacrimal sac fossa is formed by the anterior lacrimal crest of the frontal process of the maxillary bone and the posterior lacrimal crest of the lacrimal bone. It may appear radiolucent, indicative of a lacrimal sac filled with fluid or tear, and may appear clear when air-filled. 

**Figure 1:**
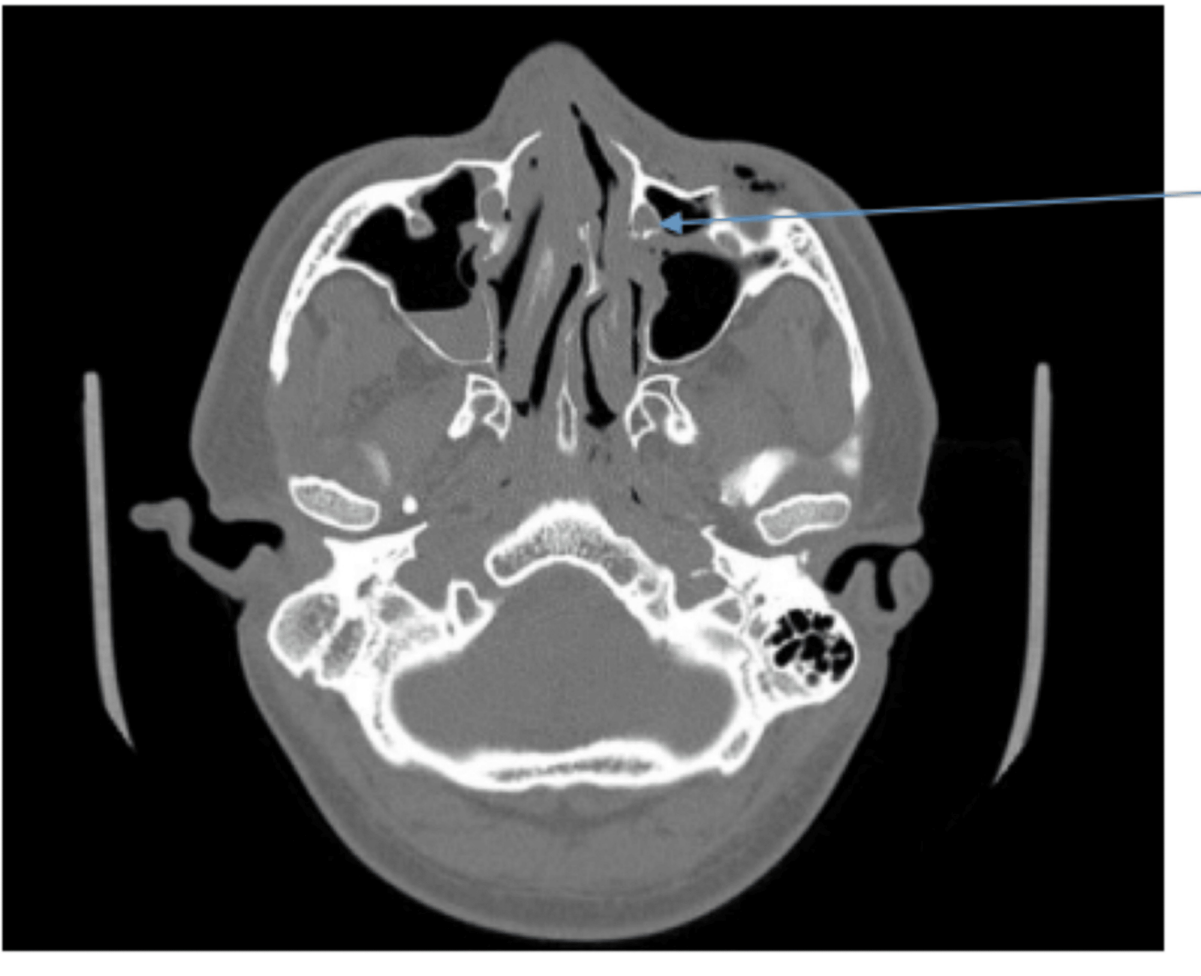
The axial section of a computed tomography scan of a fronto-naso-orbito-ethmoid fracture shows a collapse in the contour of the bony nasolacrimal canal. Arrow points out the bony nasolacrimal canal in axial view.

The CT was viewed, and the nasolacrimal canal was visualised in the axial section as per the bony landmarks in the Radiant DICOM Viewer (Medixant, Poznań, Poland) (Figure 1). The entire sequence of the axial section was thoroughly checked to assess the contour of the bony canal. For each CT, any discontinuity or collapse was noted. The area of maximum collapse was noted and marked.

**Figure 2:**
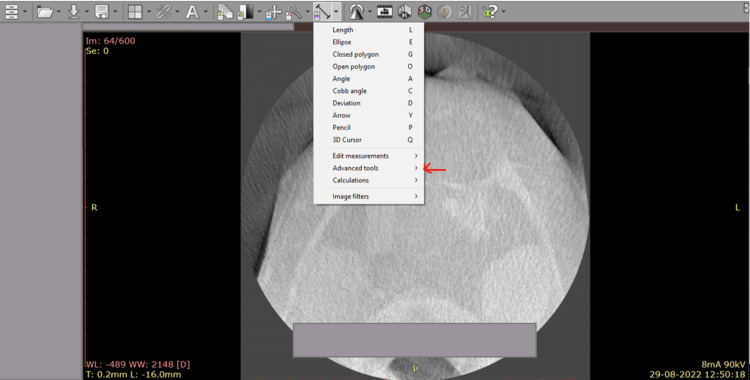
Radiant DICOM software shows settings and options for measurement of the bony nasolacrimal canal. The advanced tools option helps in the appropriate assessment of the bony nasolacrimal canal.

**Figure 3:**
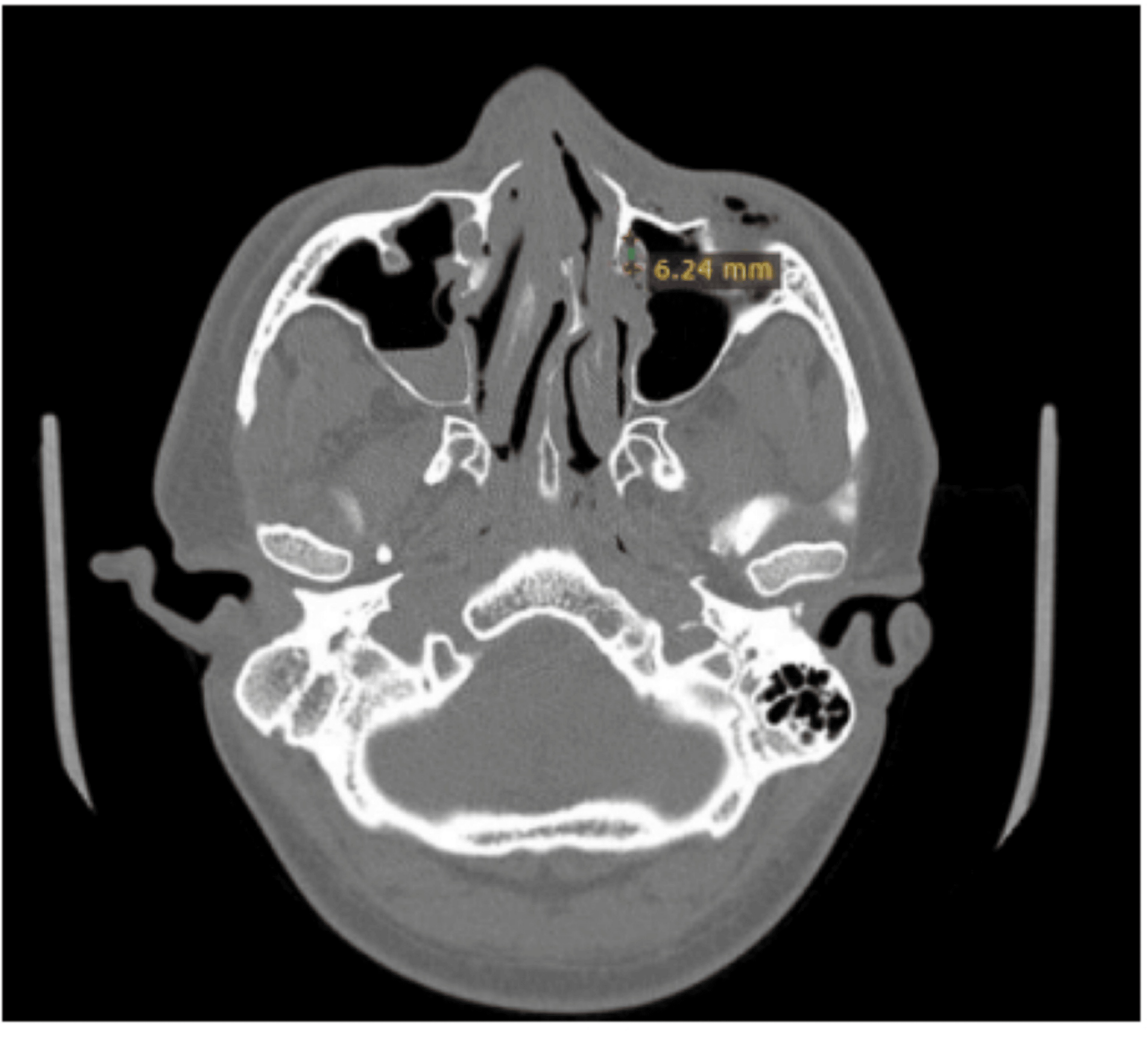
The axial section of the computed tomography scan of the fronto-naso-orbito-ethmoid fracture shows calibrated measurements using advanced tools to measure the collapse in the bony nasolacrimal canal on the radiographically assessed fracture site.

The section was marked using the measurement tool available on the toolbar. The measurements were calibrated and assessed using the advanced tools option (Figure 2). It was tabulated in millimetres (Figure 3). In cases of unilateral involvement, the comparison was made with the contralateral normal side. In the case of bilateral NLA involvement, the extent of involvement and loss of bony contour was noted and measured. These measurements were tabulated. 

**Figure 4:**
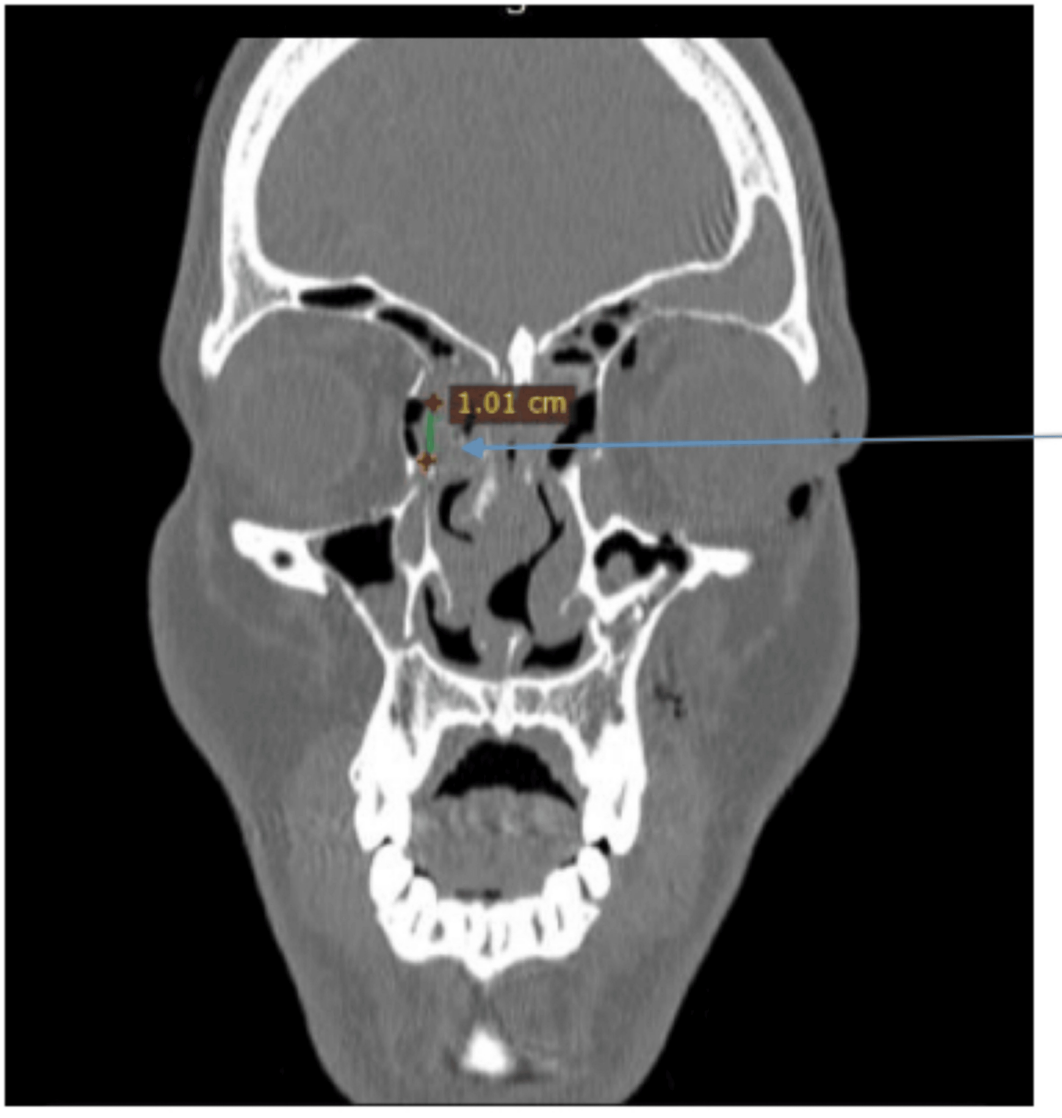
The coronal section of the computed tomography of a fronto-naso-orbito-ethmoid fracture shows the measurement of the disrupted portion of the bony nasolacrimal apparatus. The arrow showcases the measured segment in millimetres and the view of the bony nasolacrimal canal.

In the coronal section, the nasolacrimal apparatus was visualised from the bony prominence along the attachment of the medial canthal ligament, and the presence of any disruption or collapse was noted. Using the above-mentioned tools, the length of disruption was measured (Figure 4). Sagittal sections were used to confirm axial and coronal section findings. Once the involvement of the bony nasolacrimal apparatus was confirmed in all three sections, they were documented as avulsion, communication, and linear fractures.

## Results

This CT study revealed that of the total number of fractures, 121 patients out of the total 322 patients showed involvement of NLA, indicating an incidence of 37.6%. Maximum fractures seen were ZMC fractures, wherein the ZMC fractures, which are not pure zygomatic fractures, have been included. They were concomitant with fractures having medial extension. In terms of injuries, avulsion of the fossa was seen in 2.5% (eight out of 322 fractures); communication of the fossa or canal in 2.8% (nine out of 322 fractures); and linear fracture of the canal in 32.0% (103 out of 322 fractures) CTs (Table 1).

**Table 1:** Overview of demographic data, types of fractures, involvement of nasolacrimal apparatus, and types of injury to nasolacrimal apparatus in the 322 computed tomography scans of facial bones

Overview of the types of injury		Count	Column N %
Age group (in years)	<20	14	4.3%
	21-30	64	19.9%
	31-40	78	24.2%
	41-50	70	21.7%
	51-60	42	13.0%
	61-70	24	7.5%
	>71	30	9.3%
Gender	Females	78	24.2%
	Males	244	75.8%
Aetiology	Assault	34	10.6%
	Fall	56	17.4%
	Road traffic accident	232	72.0%
Type of fracture	Zygomaticomaxillary complex	228	70.8%
	Nasal bone	44	13.7%
	Le fort I	20	6.2%
	Le fort II	8	2.5%
	Le fort III	4	1.2%
	Naso-orbito-ethmoidal	6	1.9%
	Fronto-naso-orbito-ethmoidal	12	3.7%
	Frontal bone	0	0.0%
Nasolacrimal apparatus involvement	Not involved	201	62.4%
	Involved	121	37.6%
Types of injuries to nasolacrimal apparatus	No injury/displacement	202	62.7%
	Avulsion of fossa	8	2.5%
	Communication of fossa/canal	9	2.8%
	Linear fracture of the canal	103	32.0%

Statistical analysis was carried out, wherein the Kruskal-Wallis test was used to identify the relation of type of fracture and type of injury to NLA. The Mann-Whitney U test was used to assess involvement.

Types of injuries to NLA can be assessed as no injury/displacement, avulsion injuries to the fossa, communication of the fossa or canal, and linear fractures of the canal.

The axial section showed statistical significance in the involvement of NLA (Table 2), which was statistically significant with a p-value <0.0001. The fractures having at Le Fort III fractures with a median of 2.15 mm, NOE fractures with a median of 0.90 mm, and fronto-naso-orbito-ethmoidal (FNOE) fractures with a median of 1.15 mm with percentiles of 4.30, 1.10, and 1.30, respectively, and the p-value of which was 0.002%. Of these, the avulsion of the fossa had a median mm of 1.05mm, communication of the fossa or canal with a median of 1.30 mm, and linear fracture of the canal measured a median of 2.70 mm.

**Table 2:** Statistical significance of the level of canal involvement measured in millimetres in different fracture types and injuries to nasolacrimal apparatus in the axial view of the computed tomography scans

Axial view					p-value
		Median	Percentile 25	Percentile 75	
Type of fracture	Zygomaticomaxillary complex	0.00	0.00	2.60	0.002
	Nasal bone	0.00	0.00	0.90	
	Le fort I	0.00	0.00	0.00	
	Le fort II	0.00	0.00	0.85	
	Le fort III	2.15	0.00	4.30	
	Naso-orbito-ethmoidal	0.90	0.50	1.10	
	Fronto-naso-orbito-ethmoidal	1.15	0.45	1.30	
Nasolacrimal apparatus involvement	Not involved	0.00	0.00	0.00	<0.0001
	Involved	2.60	1.30	3.10	
Types of injuries to nasolacrimal apparatus	No injuries	0.00	0.00	0.00	<0.0001
	Avulsion of fossa	1.05	0.75	3.77	
	Communication of fossa /canal	1.30	0.90	1.30	
	Linear fracture of the canal	2.70	2.20	3.10	

The coronal section shows statistical significance in the involvement of NLA (Table 3) with a p-value <0.0001. The fractures had a median of 7.00 mm. The type of injuries that showed avulsion of the fossa had a median of 9.00 mm; communication of the fossa or canal showed 6.52 mm; and linear fracture of the canal showed a median of 7.00 mm. There was statistical significance in the above values with a p-value <0.0001.

**Table 3:** Statistical significance of the level of canal involvement measured in millimetres in different fracture types and injuries to nasolacrimal apparatus in the coronal view of the computed tomography scans

Coronal view					p-value
		Median	Percentile 25	Percentile 75	
Type of fracture	Zygomaticomaxillary complex	0.00	0.00	7.00	<0.0001
	Nasal bone	0.00	0.00	7.00	
	Le fort I	0.00	0.00	0.00	
	Le fort II	0.00	0.00	3.00	
	Le fort III	3.26	0.00	6.52	
	Naso-orbito-ethmoidal	9.00	8.00	10.00	
	Fronto-naso-orbito-ethmoidal	4.50	1.50	5.00	
Nasolacrimal apparatus involvement	Not involved	0.00	0.00	0.00	<0.0001
	Involved	7.00	6.00	8.00	
Types of injuries to nasolacrimal apparatus	No injuries	0.00	0.00	0.00	<0.0001
	Avulsion of fossa	9.00	4.50	10.00	
	Communication of fossa or canal	6.52	5.00	8.00	
	Linear fracture of the canal	7.00	6.13	8.00	

## Discussion

Bony nasolacrimal fossa and canal injuries are not described much in literature in patients with facial trauma [1-3]. Characterisation of these injuries may help maxillofacial surgeons better predict the requirements of surgical intervention for patients.

Unger et al. found that NLA fractures occur in association with unilateral facial fractures and with more complex fractures of the midface [1]. Three kinds of nasolacrimal fractures were identified: avulsion of the fossa, comminution of the fossa or canal, and linear fractures of the canal. The majority of the fractures of the nasolacrimal canal were comminution of the fossa and canal. Complications related to injury to the NLA were noted. Garg et al. found that 104 patients with NLA fractures among 1,980 patients with craniofacial trauma had an approximately 10% risk of developing epiphora or dacryocystitis [2]. Five NLA fracture findings were significantly associated with the development of lacrimal outflow obstruction, with the presence of nasomaxillary buttress fracture and displacement suggesting a significantly higher risk of needing lacrimal surgery. Nykamp et al. and Markowitz et al. both found a strong correlation between facial injuries and injuries to the nasolacrimal canal [3,4]. In our study, the correlation between the soft tissue injury in the midface and the nasal injury was also found. This suggests the importance of soft tissue consideration of the hard tissue in cases of midface fracture.

Stranc et al. presented the results of the primary treatment of eight patients with naso-ethmoid injury and an associated traumatic pseudo hypertelorism. The results showed that with appropriate planning, near-normal anatomy with fewer complications could be achieved [5].

Nasolacrimal canal fractures are a common complication of facial trauma, occurring in up to 15% of cases [6]. Several studies have investigated the clinical implications of nasolacrimal canal fractures. In a study, Gruss et al. found that nasolacrimal injuries were associated with NOE fractures, with a higher incidence of epiphora and dacryocystitis compared to other types of facial fractures [7]. External compression of the nasolacrimal system may occur due to malpositioned bone fragments and segments. Obstruction usually occurs in the bony nasolacrimal canal. Hence, the importance of identification of NLA and the type of injury for adopting an appropriate treatment protocol has been stressed in the literature. Similarly, it was reported that assessment of medial orbital wall fractures could help assess the NLA [8].

Management of nasolacrimal fractures can be challenging and requires a multidisciplinary approach. In a review article, Linberg and McCormick emphasised the importance of close collaboration between ophthalmologists, otolaryngologists, and maxillofacial surgeons in the management of these injuries [9].

A study by Balaji elaborated on nasolacrimal duct injuries during surgical interventions like midfacial advancements [10]. A study by Moubayed et al. evaluated the incidence of nasolacrimal canal fractures in 436 patients with maxillofacial fractures. They found that 30.7% of patients had nasolacrimal canal fractures, with the majority of these occurring in combination with other midfacial fractures. This study also found that the presence of a nasolacrimal canal fracture was significantly associated with epiphora and dacryocystitis [11].

Another study by Lee et al. [12] investigated the outcomes of patients with nasolacrimal canal fractures who underwent surgery. The study included patients who underwent dacryocystorhinostomy (DCR) for nasolacrimal canal obstruction caused by a fracture over a period of 10 years. The authors reported a majority of patients experienced resolution of epiphora and dacryocystitis.

In terms of risk factors for nasolacrimal canal fractures, a study by Poh et al. identified various risk factors. The study included 300 patients with maxillofacial fractures, of which 21% had nasolacrimal canal fractures [13].

Finally, a study by Penttila et al. investigated the use of endoscopic dacryocystorhinostomy (EnDCR) for the treatment of nasolacrimal canal obstruction caused by fractures. An EnDCR is an effective procedure in adult patients with saccal and postsaccal obstructions of the lacrimal pathway. The study stresses on appropriate with appropriate pre- and postoperative assessments [14].

The previous studies have enlisted the need for soft tissue injury and its appropriate management in midface fractures, especially in relation to NLA. In our study, we have documented various parameters and aspects of NLA injuries and their identification on a CT. Our study primarily focused on the incidence and characteristics of nasolacrimal canal fractures and the potential clinical implications of these injuries.

Further patient follow-up in the departments of ophthalmology and otolaryngology has shown the significant impact of NLA obstruction on a patient's quality of life and surgical intervention.

Therefore, it is important for clinicians to be aware of the potential for these clinical outcomes and to take steps to prevent and treat them when necessary. Future studies exploring the relationship between nasolacrimal fractures and clinical symptoms may help us better understand the underlying mechanisms and risk factors for these complications.

It is important to note that the management of nasolacrimal fractures requires a multidisciplinary approach such that ophthalmologists, otolaryngologists, and maxillofacial surgeons can help ensure optimal outcomes for patients with these injuries [15,16].

Literature has proved the successful role of CT as the most effective technique for defining the extent and nature of diseases of the nasal cavity, paranasal sinuses, and orbit [17-19]. The relatively simple clinical techniques for the diagnosis of diseases affecting the lacrimal drainage apparatus obviate sectional imaging in most cases of lacrimal sac obstruction [19-21].

Over the last few decades, there has been increased importance in providing customised multidisciplinary care to adults who have sustained facial injuries by providing a clinically relevant review of the role of multidetector CT in the management of each midfacial subunit [16-19]. There has been increasing emphasis on the surgical anatomy of soft tissues and structures, important diagnostic CT findings and their management, and common post-traumatic and postoperative complications [14,19]. 

This study is mainly based on the correlation of radiologically diagnosed fractures with their impact on the nasolacrimal canal, wherein the main focus is on midface fractures.

This study can be considered an epidemiological study that has aimed to explain the increased incidence of such injuries by radiographic imaging. Our study does not include complete clinical correlation, which may be considered a limitation of this study. Hence, in the future, it could be considered as a basis to proceed with a clinically correlated study.

Another limitation could be a lack of discussion on radiographic diagnosis and the resultant choice of apt management of these injuries. This study has formed a basis for the authors to work towards a prospective study relating the incidence of nasolacrimal injury to clinical presentations, thereby proposing a protocol for exploring nasolacrimal apparatus injuries.

## Conclusions

Injury to the NLA causes impaired lacrimal flow, inflammation, infection, obstruction of the duct, epiphora, dacrocystitis, and mucocele. Out of the CT scans of patients with maxillofacial fractures, the NLA involvement had an incidence of 37.6%. The NLA is not commonly addressed when viewed in a CT and has not been discussed in detail.

Furthermore, the postoperative review should include not only clinical presentations but also radiological examination for better results. Previous studies have explained a classification based on CT-anatomy of the NLA; this study proves the requirement of soft tissue management and clinical correlation of such injuries for earlier and apt management of the bony and soft tissue injuries of NLA.
